# Effects of Pranayama on Cancer Patients: A Narrative Review of Clinical Outcomes

**DOI:** 10.7759/cureus.54688

**Published:** 2024-02-22

**Authors:** Selvaraj Giridharan, Bhuvana Pandiyan, Nagaraj V Kumar, Soni Soumian

**Affiliations:** 1 Oncology Department, Tawam Hospitals, Al Ain, ARE; 2 Psychiatry Department, Worcestershire and Herefordshire Health and Care NHS Trust, Hereford, GBR; 3 Emergency Department, Tawam Hospital, Al Ain, ARE; 4 General Surgery Department, University Hospitals of North Midlands NHS Trust, Stoke-on-Trent, GBR

**Keywords:** qol, well being, cancer, yogic breathing, pranayama

## Abstract

Pranayama, a set of yogic breathing techniques, is being studied as a potential supportive care option for cancer patients. This review intends to evaluate the effectiveness of Pranayama in enhancing the quality of life and well-being of cancer patients based on evidence from randomized controlled trials.

We thoroughly searched databases for studies published between 2013 and 2023. We focused on randomized controlled trials that compared Pranayama interventions with non-yoga control groups. We assessed the primary outcomes, including psychological well-being, quality of life, physiological parameters, and treatment-related side effects.

The review indicates that practicing Pranayama can lead to significant improvements in antioxidant levels, stress, anxiety, sleep quality, and overall quality of life for cancer patients. However, the evidence comes from a limited number of studies, which vary in sample sizes and methodologies.

Pranayama shows promise as a complementary therapy for cancer patients, potentially enhancing their well-being and quality of life. Nonetheless, the current evidence base is limited, necessitating further research with larger sample sizes and more rigorous study designs to confirm these findings and elucidate the underlying mechanisms.

## Introduction and background

Introduction

Cancer is a multifaceted disease characterized by abnormal cell growth with the potential to invade or spread to other parts of the body. The incidence of cancer is on a rising trajectory, with the World Health Organization reporting an estimated 19.3 million new cancer cases and almost 10 million cancer-related deaths in 2020 [1]. This increasing trend is anticipated to escalate, with projections suggesting 28.4 million cases by 2040, a surge of 47% from 2020. This alarming rise is partly attributed to population growth and aging, as well as changes in the prevalence and distribution of significant risk factors for cancer, many of which are associated with socioeconomic development. According to the World Health Organization, Asia has reported high cancer incidence and death rates, with statistics indicating 48.4% and 57.3%, respectively [2].

The diagnosis and treatment of cancer can have significant physical and psychological effects on patients, resulting in stress, anxiety, and various side effects associated with conventional therapies. That is why it is crucial to incorporate supportive care methods that complement conventional medical treatments [3,4]. Complementary approaches, such as Pranayama, provide a holistic avenue for mitigating some of these burdens. These practices foster relaxation, improve mental well-being, and enhance overall quality of life, playing a crucial role in the comprehensive care of cancer patients.

The integration of complementary therapies into cancer care not only addresses the multifaceted impacts of the disease but also empowers patients in their healing process [5]. This highlights the value of holistic health practices in modern healthcare and underscores the importance of considering them as an integral part of cancer care. Such an approach can provide comprehensive patient care and improve overall health and well-being.

Pranayama, an integral aspect of yoga, is one of the ancient Indian practices that transcended its traditional boundaries to gain global recognition for its health benefits. The concept of Pranayama is deeply embedded in the philosophical and practical framework of Yoga, as outlined in key texts like 'Patanjali's Yoga Sutras [6]. Historically, Pranayama was more than just a practice; it was a way of life that emphasized the balance and connection between the body, mind, and spirit.

The term "Pranayama" is derived from two Sanskrit words: "prana,” which signifies the vital life force that pervades all living entities, and "yama,” which means control or extension. This practice involves a series of breathing exercises designed to control rhythm and depth of breath [7]. It is believed that by controlling breath, one could influence the flow of prana in the body, thus enhancing vitality and mental focus.

Pranayama uses various techniques, including Nadi Shodhana, which aims to harmonize the left and right hemispheres of the brain, thereby fostering mental clarity and calmness. Kapalabhati is a stimulating practice that enhances lung capacity and invigorates the mind. Conversely, Bhramari is known for its soothing effect, which is particularly useful for reducing stress and anxiety. Sudarshan Kriya is another technique that includes Ujjayi breathing and Bhastrika and concludes with Om chanting and silence, leading to a deep meditative state.

These practices vary in approach, some focus on calming the mind while others energize the body or aid in cleansing and detoxification processes. Physiologically, Pranayama influences the autonomic nervous system, which controls automatic bodily functions such as heartbeat and digestion. Regular practice is believed to improve the balance between the sympathetic and parasympathetic systems, which is crucial for maintaining physical and mental health, particularly for managing stress and anxiety [8].

In addition to its physiological impact, Pranayama is often praised for its psychological benefits. This is a powerful tool for enhancing mental clarity, concentration, and emotional stability [9]. By regulating breath, Pranayama practitioners claim to achieve inner peace and mindfulness, which can be particularly beneficial in managing life's stresses and strains [10].

Pranayama, with its potential to improve the quality of life and manage treatment-related side effects, holds a unique position in cancer care. The physical and psychological challenges that often accompany cancer treatment can be daunting. However, Pranayama's ability to alleviate stress, improve respiratory function, and enhance overall well-being could offer a supportive care approach that complements traditional cancer treatments, making it particularly beneficial for patients with cancer [11-13].

The therapeutic benefits of Pranayama have been extensively studied. These studies have revealed various physiological and psychological advantages of pranayama. Physiologically, Pranayama has been shown to enhance cardiorespiratory fitness, improve cardiovascular and respiratory functions, and increase parasympathetic tone while decreasing sympathetic activity [14-17]. Furthermore, Pranayama has been found to have positive effects on various respiratory and cardiovascular conditions such as asthma, chronic obstructive pulmonary disease (COPD), and hypertension [18].

Pranayama has been found to provide a range of psychological benefits, making it a recommended low-risk and low-cost adjunct to conventional treatments for stress-related medical conditions, substance abuse, and criminal offender rehabilitation [19]. Studies have shown that Pranayama can improve mental quality of life, sleep disturbance, and overall wellness [20,21]. Additionally, it has been suggested to enhance affect and reduce stress, making it a potential complementary therapy for smoking cessation and stress-related conditions such as combat stress in military personnel [22].

In addition to its physiological and psychological benefits, Pranayama has been integrated into comprehensive yoga practices, including physical postures, meditation, and relaxation techniques. This multifactorial approach is practical for managing conditions such as knee arthritis, osteoarthritis, and diabetes, as well as improving functional disability, pain, and flexibility in osteoarthritis of the knee joint [23,24]. Furthermore, Pranayama has been associated with improved biopsychosocial changes in older adults with lower-extremity osteoarthritis, highlighting its potential to promote overall well-being.

Overall, the practice of Pranayama embodies a holistic approach to health, viewing the individual as an integrated whole. In the modern healthcare context, where there is a growing appreciation for the interplay between mind and body, Pranayama offers a time-tested, non-invasive approach to enhancing well-being, which may be particularly valuable in managing complex conditions such as cancer. While there have been several studies on the impact of Pranayama in cancer patients, no reviews have been conducted to date.

Objective

This review aimed to evaluate the effects of Pranayama on cancer patients. The focus is on its influence on cancer-related symptoms, treatment side effects, quality of life, psychological well-being, and physiological outcomes. The motivation for this review arises from the increasing interest in complementary and alternative medicine (CAM) for cancer management, given the challenges that patients face with conventional treatments [25,26]. Pranayama is being considered as an adjunct therapy in cancer care due to its stress-reducing and well-being-enhancing properties. This review intends to examine the evidence for the effectiveness and safety of Pranayama in cancer patients to clarify its potential role, inform clinical practices, and for further research in oncology.

Methods

This narrative review presents a methodological approach combining systematic search elements with flexibility in the flexibility of narrative synthesis. Our search was conducted on January 2024 and spanned several databases, namely, PubMed, Cochrane Library, Web of Science, Amed, Embase, Emcare, and Cinahl, targeting English-language randomized controlled trials published between January 2013 and December 2023. We focused on studies involving 'Sudarshan Kriya,' 'Pranayama,' and 'breathing exercises' in cancer care and filtered for quality evidence.

Our criteria for inclusion were trials examining Pranayama's physiological and psychological effects on cancer patients while excluding non-randomized studies, commentaries, and those lacking primary data or relevant outcomes. Titles and abstracts were screened to identify relevant studies, with discrepancies resolved through team discussions. We extracted data on study design, sample size, interventions, and outcomes, which were narratively synthesized to reflect Pranayama's impact on cancer care.

**Figure 1:**
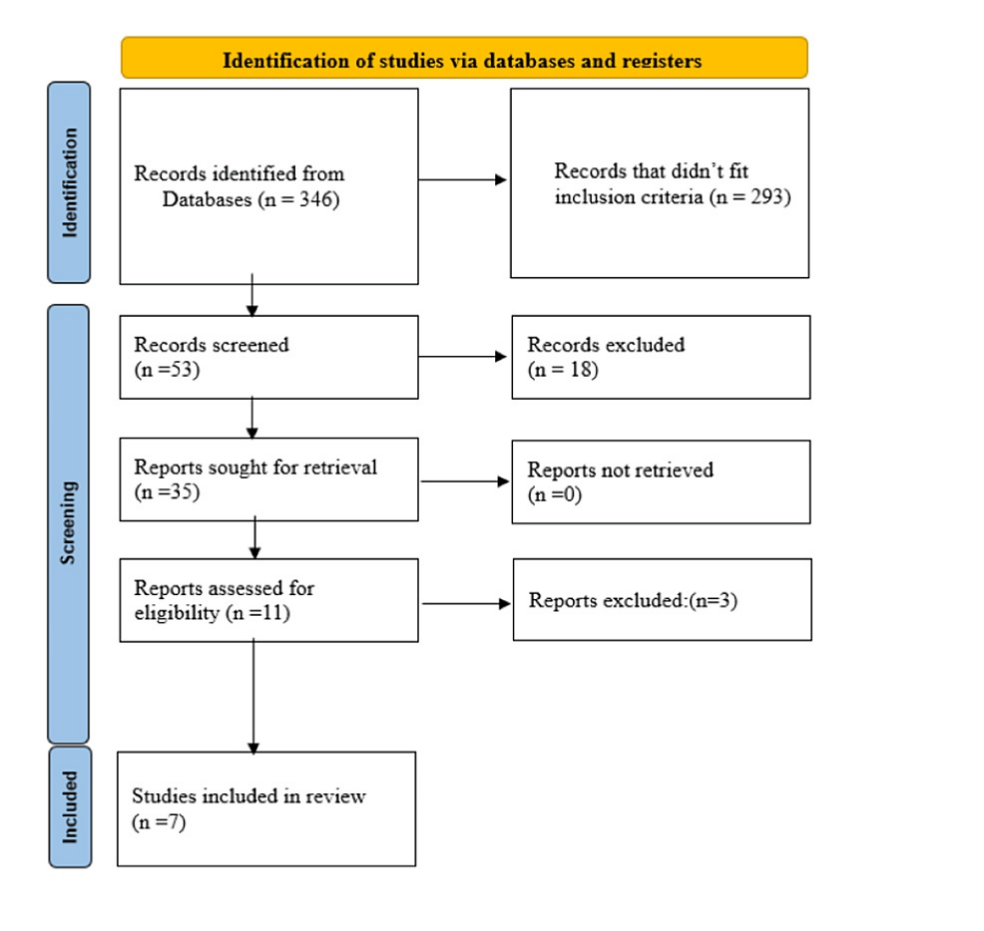
Summarized search strategy

Although not strictly adhering to Preferred Reporting Items for Systematic Reviews and Meta-Analyses (PRISMA) guidelines, this methodological approach ensures a rigorous yet flexible review suitable for capturing the broad evidence spectrum on Pranayama in cancer patient care (Figure 1) The review highlights the need for further research to establish the effectiveness of Pranayama in cancer care, especially in alleviating physical and psychological symptoms and improving quality of life.

## Review

Results

Our search returned 346 publications that could be relevant to our research. After eliminating publications that did not meet our inclusion criteria and removing duplicates, 53 publications remained. We then screened these publications based on their titles and abstracts using specific inclusion criteria, which helped us further narrow the selection to 35 studies. After reviewing the full texts of these 35 studies in detail, we shortlisted 11 articles. Finally, we found that only seven of these studies met all the criteria for inclusion in our final review, as shown in Figure 1.

Herein, we present a detailed analysis of the seven selected studies and summarize their key findings in Table 1.

**Table 1:** Summary characteristics of the studies included in the review

Author and Year	Sample Size	Study Aim	Methods and Patient Selection	Conclusion
Kumar et al. 2013 [27].	Total 147 (Pranayama group n=78, Control group, n=69)	To evaluate the effects of Sudarshan Kriya and Pranayama on serum cortisol levels and pain perception among women with advanced-stage breast cancer.	One hundred and forty-seven participants were randomly assigned to either a standard care group or an intervention group receiving SK and P intervention in addition to standard care. The intervention involved an 18-hour workshop followed by daily practice at home.	The intervention group showed a significant reduction in serum cortisol levels and pain perception, indicating decreased stress levels and effective pain alleviation.
Chakrabarty J et al. 2013 [28].	Total 160 (Pranayama group n=80, Control group, n=80)	To investigate the effect of Pranayama on protein thiols and glutathione levels in breast cancer patients undergoing radiation therapy.	One hundred and sixty patients were randomly assigned to either an experimental group receiving Pranayama and radiation therapy or a control group receiving only radiation therapy. Pranayama was practiced daily for six weeks.	Pranayama may help maintain or enhance the antioxidant defense system, indicated by increased protein thiol and glutathione levels in the experimental group.
Chakrabarty J et al. 2015 [29].	Total 160 (Pranayama group n=80, Control group, n=80)	To evaluate the impact of Pranayama on cancer-related fatigue in breast cancer patients undergoing radiation therapy.	Same cohort as the 2013 study.	Pranayama practice resulted in a significant difference in cancer-related fatigue levels, with the experimental group experiencing less severe symptoms of fatigue.
Chakrabarty J et al. 2016 [30].	Total 160 (Pranayama group n=80, Control group, n=80)	To assess the impact of Pranayama on emotional aspects such as impatience, worry, anxiety, and frustration in the same cohort.	Same cohort as the 2013 study.	Pranayama significantly reduced worry, anxiety, and frustration in the experimental group, suggesting improved emotional well-being.
Lu et al. 2023 [31].	Total 108 (Pranayama group n=36, Pranayama and Problem-solving group=36, Control group, n=36)	To investigate the effectiveness of yoga breathing exercises and a problem-solving model in improving perioperative outcomes for lung cancer patients.	One hundred and eight lung cancer patients were randomly assigned to one of three groups: yoga breathing exercises with a problem-solving model, yoga breathing exercises alone, or usual care. Outcomes were evaluated at multiple points.	The combined intervention of yoga breathing exercises and the problem-solving model showed significant improvements in dyspnoea, exercise capacity, and anxiety levels.
Kaur et al. 2023 [32].	Total 84 (Pranayama group n=42, Control group, n=42)	To examine the effectiveness of Pranayama in reducing fatigue among cancer patients undergoing External Radiation Therapy (ERT).	Eighty-four cancer patients were randomly assigned to an experimental group receiving Pranayama or a control group. Fatigue levels were evaluated using the Cancer Fatigue Scale.	Pranayama significantly reduced fatigue levels in the experimental group, with a notable mean difference in fatigue levels post-intervention.
Joshi et al. 2023 [33].	Total 27 (Pranayama group n=12, Control group, n=15)	To examine the effectiveness of Pranayama and mindfulness meditation in managing emotional distress and fatigue among hematological cancer patients.	Twenty-seven adult patients were randomized into an intervention group receiving daily sessions of Pranayama and mindfulness meditation or a control group. The effectiveness was evaluated using the Emotion Thermometer and the Fatigue Assessment Scale.	The intervention group showed a significant reduction in emotional distress, anxiety, anger, need for help, and fatigue compared to the control gr

Kumar et al. conducted a randomized controlled trial to evaluate the effects of Sudarshan Kriya (SK) and Pranayama (P) on serum cortisol levels and pain perception in women with advanced-stage breast cancer [27]. One hundred and forty-seven participants were randomly assigned to either a standard care group (n=69) or an intervention group receiving standard care along with the SK and P interventions (n=78). The intervention involved an 18-hour workshop spanning three days, focusing on self-awareness, Ujjayi breath, Bhastrika Pranayama, and rhythmic breathing associated with SK, under the guidance of trained yoga teachers. After the workshop, participants in the intervention group were instructed to practice these techniques at home for 20 minutes daily in conjunction with their standard pharmacological treatment for pain, with a family member assigned to monitor their regular practice. The results after three months of practice showed that the intervention group had a significant reduction in serum cortisol levels compared to the control group, indicating a decrease in stress levels among those practicing SK and P. Specifically, cortisol levels in the intervention group were significantly lower at both the third and sixth-month visits, demonstrating sustained stress reduction over time. Pain perception also showed notable improvements in the intervention group, with a reduction of three points on the pain scale at both the third and sixth-month visits, suggesting that SK and P could effectively alleviate pain in patients with advanced-stage breast cancer.

In 2013, Chakrabarty et al. conducted a randomized controlled trial (RCT) to investigate the effect of Pranayama on protein thiols and glutathione levels in breast cancer patients undergoing radiation therapy [28]. This study evaluated whether Pranayama could prevent the reduced antioxidant levels that usually occur following radiation therapy. A total of 160 breast cancer patients were randomly assigned to either an experimental group (n=80) that received Pranayama along with radiation therapy or a control group (n=80) that received only radiation therapy with routine care. The Pranayama intervention involved three specific techniques: Nadi Sodhana, Sheethali, and Brahmari, with a daily practice time of approximately 18 minutes in the morning and evening for six weeks. Blood samples were collected from both groups at the beginning and end of the six-week treatment period. The study findings showed no significant difference in protein thiol levels between the two groups before RT. However, after the completion of radiation therapy, a significant increase in protein thiol levels was observed in the experimental group as compared to that in the control group. This suggests that Pranayama may help to maintain or enhance the antioxidant defense system in patients with breast cancer undergoing radiation therapy. The mean glutathione levels were also higher in the experimental group than in the control group, indicating a potential reduction in oxidative stress among those practicing Pranayama. These results suggest that Pranayama may protect against oxidative stress induced by radiation therapy, possibly by enhancing the body's antioxidant defenses.

The same group of researchers reported another outcome measure of cancer-related fatigue in the same cohort of patients two years later [29]. They used the Cancer Fatigue Scale, which measures fatigue across physical, functional, affective, and cognitive domains. The scale classifies fatigue severity into four levels: no fatigue, mild fatigue, moderate fatigue, and severe fatigue. At the beginning of the study, most participants in both the experimental (76.25%) and control (87.5%) groups reported only mild fatigue. A small percentage of patients in both groups reported severe fatigue (experimental group: 11.25%, control group: 8.75%). Upon completion of radiation therapy, the data revealed a significant difference in cancer-related fatigue levels between the two groups. Patients in the control group, who received only radiation therapy, experienced more severe symptoms of fatigue than those in the experimental group who practiced Pranayama.

A year later, in 2016, the same group reported another outcome measure of emotional aspects such as impatience, worry, anxiety, and frustration [30]. The assessment of emotional aspects is an integral part of evaluating cancer-related fatigue, utilizing a scale with five items, each rated on an 11-point scale (0-10, with 0 indicating the least negative emotion). The items specifically measured the degree of tiredness, impatience, worry, anxiety, and frustration experienced by patients. The content validity of the scale was ensured through an expert review. Patients were surveyed at the beginning of their radiation therapy and again six weeks after completion of their treatment. The findings revealed that emotional distress was prevalent among the patients, with worry being the most reported negative emotion, demonstrating a significant difference in the emotional well-being of the two groups at the end of the treatment period. Patients in the experimental group who practiced Pranayama reported significantly lower levels of worry, anxiety, and frustration than those in the control group, and had lower mean scores for negative emotions. Interestingly, the study observed no significant change in the emotional scores of the control group, suggesting that without an intervention such as Pranayama, emotional distress associated with cancer treatment might remain unmitigated.

Lu et al. conducted a randomized controlled trial to investigate the effectiveness of yoga breathing exercises and a problem-solving model in improving perioperative outcomes in adults with lung cancer undergoing surgical resection [31]. This study aimed to address the significant burden of lung cancer and its surgical treatment on patients, proposing yoga breathing exercises as a viable method for pulmonary rehabilitation to enhance patients' recovery and well-being. The study enrolled 108 lung cancer patients randomly assigned to one of three groups: one group received yoga breathing exercises based on a problem-solving model, another group received only yoga breathing exercises, and the third group received usual care. The primary outcome measures included dyspnoea, exercise capacity, anxiety, depression, and postoperative indwelling time of the thoracic drainage tube, along with patient compliance. These outcomes were evaluated at multiple time points: upon admission, the day before surgery, and at discharge.

The results indicated that patients in the group receiving the combined intervention of yoga breathing exercises and the problem-solving model experienced significantly greater improvements in dyspnoea, exercise capacity, and anxiety levels than those in the control group. Specifically, yoga breathing exercises alone were practical in significantly reducing patients' dyspnoea and anxiety. When comparing the two intervention groups, a notable difference was observed in exercise capacity and compliance, favoring the combined approach of yoga breathing and the problem-solving model. However, the study found no significant differences among the three groups in terms of depression and indwelling time of the thoracic drainage tube.

The trial conducted by Kaur et al. examined the effectiveness of Pranayama in reducing fatigue among cancer patients undergoing External Radiation Therapy (ERT) [32]. The sample consisted of 84 cancer patients randomly assigned to either an experimental or control group, with an equal distribution of 42 participants in each group. The Cancer Fatigue Scale (CFS) developed by Toru Okuyama was used to evaluate fatigue levels. The experimental group received Pranayama intervention consisting of various breathing techniques, including Sahaj Pranayama, Nadi Sodhana, Sheethali, Brahmari, Ujjayi, Chandrabhedi, Suryabhedi, and Bhastrika, for 40 min daily over 12 days. The study findings indicated a significant reduction in fatigue levels among the experimental group participants post-intervention. Specifically, 97.6% of participants in the experimental group reported mild fatigue while 64.3% of participants in the control group experienced severe fatigue. The statistical analysis revealed a significant mean difference in fatigue levels between the two groups post-intervention (t=17.99, df=41, p-value<.001), highlighting the efficacy of Pranayama in reducing fatigue among ERT recipients.

The study conducted by Joshi et al. examined the effectiveness of Pranayama and mindfulness meditation in managing emotional distress and fatigue among adult hematological cancer patients undergoing chemotherapy [33]. The study involved randomizing 27 adult patients into an intervention group (n=12) or a control group (n=15). The intervention group was subjected to daily sessions of 15 minutes of slow-paced Pranayama and 15 minutes of mindfulness meditation over six weeks. The effectiveness of the interventions was evaluated using the Emotional Thermometer (ET) for emotional distress and the Fatigue Assessment Scale (FAS) for fatigue. Assessments were conducted at baseline and after the 6-week intervention period. The results demonstrated a significant reduction in emotional distress, anxiety, anger, and need for help, as well as in total, physical, and mental fatigue within the intervention group compared to the control group.

Discussion

This literature review highlights crucial findings from seven randomized controlled trials that investigated the effects of Pranayama on individuals battling cancer. The reviewed studies provide cumulative evidence that Pranayama has multifaceted benefits for cancer patients, including improvements in physiological and psychological domains. Pranayama has consistently reduced stress levels, as evidenced by decreased serum cortisol, in patients practicing Sudarshan Kriya and Pranayama alongside standard care. This reduction in stress biomarkers underscores Pranayama’s potential to enhance the body’s resilience to the psychological effects of cancer diagnosis and treatment [27].

Moreover, intervention groups demonstrated notable improvements in pain perception and antioxidant levels, especially among breast cancer patients undergoing radiation therapy. Pranayama could counteract the oxidative damage typically associated with radiation therapy, offering protection that complements conventional treatment modalities [28].

Pranayama also helps alleviate cancer-related fatigue and emotional distress, with patients reporting significant reductions in fatigue severity and negative emotions such as worry, anxiety, and frustration. These findings are consistent across various studies, indicating that Pranayama can manage the common side effects of cancer and its treatments [29,30].

Interestingly, the benefits of Pranayama extended to physical outcomes. Patients with lung cancer who engaged in Pranayama exercises exhibited enhanced exercise capacity and reduced dyspnoea, highlighting the practice’s role in supporting physical rehabilitation and recovery [31].

The various Pranayama techniques employed across the studies, from Nadi Sodhana to Bhastrika, indicate the versatility of this practice in addressing different therapeutic needs. Despite the diverse methodologies and outcome measures, the consistent observation of positive effects suggests a universal therapeutic potential of Pranayama that warrants further exploration.

Previous studies have examined the health benefits of Pranayama in various health conditions. However, our literature review focuses solely on the effects of Pranayama practices on cancer patients, which is a specialized niche within the broader scope of Pranayama research. Jayawardena et al. also highlighted the benefits of Pranayama in improving the quality of life and physiological functions in cancer patients, which aligns with our findings [34]. Kuppusamy et al. discussed the effects of Bhramari Pranayama and emphasized its potential health benefits through parasympathetic predominance [35]. However, their review indicated the need for high-quality randomized trials owing to methodological limitations in existing studies.

In contrast, our review has taken a step forward by focusing solely on randomized controlled trials, ensuring a more robust evidence base for the effects of Pranayama in cancer care. Saoji et al. provided a narrative review of yogic breath regulation and presented evidence of its beneficial effects in both physiological and clinical settings, including non-communicable and communicable diseases [36]. However, our review provides a more detailed exploration of how Pranayama addresses cancer-related symptoms and treatment side effects. Our review has taken a step forward by focusing solely on randomized controlled trials, ensuring a more robust evidence base for the effects of Pranayama in cancer care. Furthermore, our review complements this broad perspective by offering detailed evidence of Pranayama’s role in managing cancer-specific outcomes, such as symptom relief, stress reduction, and overall quality of life improvement.

To address these limitations and further our understanding of Pranayama’s role in cancer care, future research should aim for larger, multicentre trials with standardized Pranayama protocols to ensure consistency and comparability. Incorporating objective measures alongside self-reported outcomes could provide a more comprehensive assessment of Pranayama’s effects, keeping the reader abreast of the need for further research. Exploring the long-term effects of Pranayama practice and its sustainability post-intervention would offer valuable insights into its role in ongoing cancer care and survivorship. Additionally, investigating the mechanisms underlying the benefits of Pranayama could elucidate how these practices confer their therapeutic effects, potentially leading to more targeted and effective interventions.

Given the promising integration of Pranayama with other therapeutic modalities, such as mindfulness and problem-solving approaches, future studies should also explore the synergistic effects of combined interventions to optimize outcomes in cancer patients.

## Conclusions

In conclusion, Pranayama emerges as a promising complementary therapy that could significantly enhance cancer patients' well-being and quality of life. However, its application faces challenges, including potential biases from the difficulty in blinding studies and variability in practitioner delivery. The use of self-reported outcomes also risks introducing biases while the limited diversity in study populations and settings may affect the generalizability of results. To integrate Pranayama into conventional oncology and solidify its evidence base, comprehensive research with more rigorous methodologies, objective and standardized outcome measures, and a more comprehensive range of cancer populations is crucial. This approach will validate Pranayama's effectiveness and support a holistic, patient-centered cancer-care model. A call for more detailed and methodologically sound research is clear, highlighting the need to establish Pranayama's definitive role in cancer care.
